# Projecting the Target Quantity of Medical Staff in Taiwan’s Administrative Regions by the Theory of Carrying Capacity

**DOI:** 10.3390/ijerph17092998

**Published:** 2020-04-26

**Authors:** Jin-Li Hu, Ming-Chung Chang, Hsin-Jung Chung

**Affiliations:** 1 Institute of Business and Management, National Chiao Tung University, Taipei City 10044, Taiwan; 2 Department of Finance, Chihlee University of Technology, New Taipei City 22050, Taiwan

**Keywords:** data envelopment analysis, carrying capacity, carrying efficiency

## Abstract

As physicians and nurses are the main medical staff in any healthcare system, an appropriate medical workforce distribution is crucial for an aging society. This study thus applies the theory of carrying capacity and the given demand side to explore the carrying capacity, carrying efficiency, and potential adjustment ratio of medical staff in Taiwan’s administrative regions by using the data envelopment analysis (DEA) approach in which a lower carrying efficiency implies a higher shortage ratio. The main findings are as follows. (i) The carrying efficiency of Taiwan’s medical staff is weakening year by year, while the carrying efficiency of the country’s nurses is lower than that of physicians. (ii) The outlying islands of Taiwan have a more serious shortage of physicians and nurses than the main island. (iii) The central government should encourage more physicians and nurses to work in regions with a low carrying efficiency.

## 1. Introduction

In the face of the coronavirus contagion that began in December 2019, the excellent ability of epidemic prevention and control by Taiwan has been greatly noticed and applauded by countries around the world. The country set up its National Health Insurance (NHI), a government-administered insurance-based national healthcare system, in 1995. The system is characterized by comprehensive healthcare to all Taiwanese and legal residents with a low insurance payment, and collects patients’ health data to plan and improve the system. This healthcare system not only is helping the country defend itself against a short-term epidemic shock but is also confronting a long-term aged society at the same time. Hence, the carrying capacity of Taiwan’s medical system supported by NHI is valuable to study.

The World Health Organization (WHO) defines an aging society as one in which the ratio of the population over 65 years old to the total population is 7% to 14%, while an aged society is 14% to 20% for that age group, and a super senior society is that age group at 20% or greater. Data in September 2018 from the Ministry of the Interior of Taiwan show that the country is an aged society, because its population over 65 years old is 3.38 million, or 14.4% of the total population. From this viewpoint, a long-term care policy should be the key point for the Taiwan government in order to plan the proper medical work-force regional distribution, because a sufficient quantity of medical staff can help support long-term care quality.

The quantity of Taiwan’s medical workforce is increasing year by year, but the allocation of medical resources to its administrative regions exhibits a disparity. Data at the end of 2017 from the Directorate General of Budget, Accounting, and Statistics of Taiwan illustrate that the population percentage in Taipei City to the country’s total population is 11.4% but its physicians’ percentage is 20.8%, implying that there are too many medical resources concentrated in the capital city, and that there is a problem of medical resource allocation disparity among other administrative regions. Finding a solution to this problem can help improve Taiwan’s welfare.

In his book, *An Essay on the Principle Population*, Thomas Malthus in 1798 said earth could not indefinitely support an ever-increasing human population, which is the theory famously known as the carrying capacity of earth. The idea of earth’s carrying capacity is that earth has a scarcity of food, water, and so on that will limit population growth through famine if humans do not check themselves. Aside from the theory of carrying capacity by Malthus becoming an outstanding viewpoint in economics, the theory is also a well-known and widely accepted concept in ecology. The term carrying capacity can be traced to the late 1890s by managers who care about the use of land for grazing livestock (see, e.g., Bartels et al. [1]). The extensive study on carrying capacity by Godschalk and Parker [2] explores carrying capacity in natural and man-made systems. Subsequently, the United Nations Educational, Scientific, and Cultural Organization (UNESCO) and Food and Agriculture Organization of the United Nations (FAO) apply this concept to study sustainable development in Kenya (UNESCO and FAO [3]). Rundall and McClain [4] are pioneers in the concept of carrying capacity within the physician workforce literature in which they explain the physician distribution and forecast regional physician supply. Their study concludes that the density of resources causes the carrying capacity to vary, which impacts physicians’ distribution and supply. Chiang [5] applies the theory of carrying capacity to examine the location pattern of physicians in Taiwan, concluding that the attractiveness of townships is powerful in determining the physician distribution: The greater the deviation from the physician carrying capacity, the faster the growth rate of the physician-population ratio in attractive townships.

The past literature seldom combines data envelopment analysis (DEA) and the carrying capacity theory to discuss the carrying capacity of medical staff, including physicians and nurses. Our study thus not only fills this gap but also takes Taiwan as an example to compute the distribution efficiency of medical staff and to assess the target quantity of medical staff in this country’s administrative regions, which present the problem of an urban–rural disparity.

The rest of the paper runs as follows: Section 2 is the literature review; Section 3 is the methodology; Section 4 is the empirical study; and Section 5 is the conclusion and suggestions.

## 2. Literature Review

Farrell [6] was the first scholar to measure production efficiency, and his paper has inspired many studies over the years covering the best practice technology and efficiency measurement by using the DEA approach. For example, Charnes et al. [7] looked into efficiency measurement under a constant return to scale (CRS), named the DEA-CCR model, while Banker et al. [8] also examined efficiency measurement but under a variable return to scale (VRS), named the DEA-BCC model. The cornerstone of the DEA approach is linear programming, which establishes the best frontier as a criterion in order to assess the relative efficiency score of a decision-making unit (DMU), which in the DEA approach can be firms, schools, countries, hospitals, etc. Hu and Huang [9] applied the DEA-BCC model to compute hospital efficiency scores and then used both the Mann–Whitney test and Tobit regression to investigate the impacts of environmental variables on these scores. They concluded that the efficiency scores of public hospitals are significantly lower than those of private hospitals, whereas higher ward capacity utilization helps improve a hospital’s efficiency score. In addition, increasing the amount of expensive medical care equipment and beds can also significantly raise a hospital’s efficiency score.

Stone and Simmons [10] used the DEA-CCR model with health status, such as the infant mortality rate and healthy resident rate, as outputs to assess the relative efficiency of the use of physicians in Michigan counties. They also examined the relationship between the physician-population ratio and the health status of the communities they serve. Their research showed that about 47% of Michigan counties reveal effective physician usage. Chu et al. [11] examined whether the Physician Compensation Program (PCP) established in a responsibility center system could improve the departmental operational efficiency in a large Taiwan teaching hospital by using the DEA approach. They applied a Tobit regression model to examine what factors impact the departmental operational efficiency and indicated that (i) PCP implementation improves the average operational efficiency; (ii) physicians’ seniority and percentage of physicians’ service time in the department are key points of operational efficiency improvement; and (iii) departments with higher profits but fewer numbers of employees have a higher operational efficiency. Chang et al. [12] employed the DEA-CCR and DEA-BCC models to evaluate the impact of the National Health Insurance Program on district hospitals in Taiwan and concluded that the program’s implementation worsens their operating efficiency.

Kabene et al. [13] addressed the importance of human resources management in the healthcare system and further revealed how such management is essential to any healthcare system in order to improve overall patient health outcomes and to provide a high quality healthcare service. They found that high-quality healthcare depends on the proper management of human resources and the development of new policies within human resources management for healthcare. The earliest study of physicians’ demand and supply in Taiwan was by Baker and Perlman [14]. The healthcare system in Taiwan is National Health Insurance, in the United States it is Patient Protection and Affordable Care Act (PPACA), and in the United Kingdom it is National Health Service (NHS). Under these healthcare system frameworks, research on the medical workforce is the key point. Furthermore, the optimal quantity of medical staff is a major question for any medical workforce study. Tsai et al. [15] created a physician density (PD) prediction model based on a multiple stepwise-linear regression to calculate an appropriate PD number for a specific country. Their study concluded that a country with a large PD discrepancy needs to examine physicians’ workloads and their well-being. The effectiveness and efficiency of medical care can promote the population’s health and team resource management.

Physician distribution is also a critical issue in healthcare. In the late 1980s, the Japan government noticed that the shortage of doctors was becoming a social problem. Toyabe [16] compared the numbers of physicians in Japan from 1996 to 2006 and the trends in the distribution of physicians in which the Gini coefficient, Atkinson index, and Theil index were used as measures for a maldistribution of physicians to the population and used geographic information system (GIS) software to visualize the distribution of physicians on a map. This research concluded that both the shortage of physicians and the maldistribution of hospital physicians are the key problems in Japan’s healthcare system.

Early research about the distribution of physicians comes from Marden [17], who found that the factors of population size, age, race, education, and medical environment have impacted the distribution of physicians within the metropolitan areas of the United States. Chang and Halfon [18] studied the geographic distribution of pediatricians throughout the United States, indicating the phenomenon that pediatricians concentrate in states with a larger number of residency training positions and states with high per capita income. The maldistribution of pediatricians needs to be improved through a proper manpower policy. Wharrad and Robinson [19] explored the global distribution of physicians and nurses and the influence of the gross national product (GNP) per capita on this distribution by using a general regression model and found that the influence of GNP per capita on the global distribution of physicians and nurses is significant. Dussault and Franceschini [20] identified the critical determinants of the maldistribution of health personnel, concluding that equitable socioeconomic conditions between rural and urban areas, adequate investment in human resources, and stable and proper political institutions are the critical factors to achieve a balanced distribution of the health workforce. Sloan and Hsieh [21] explored nurse density by analyzing 175 nations in the world, finding a positive relationship between national income and nurse density and also providing the factors that make a difference in nurse density among countries: Gross domestic product (GDP) per capita, personal expenditure on healthcare service, and physician density. Based on past research, the optimal quantity of medical staff and a balanced distribution are two critical issues to a perfect healthcare system. We applied the theory of carrying capacity under the DEA framework and the given the demand side to find the optimal quantity and balanced distribution of the healthcare workforce. In greater detail, the assumption of the given demand side in this study reflects that the demand price and its adjustment on medical resources are ignored. One example is Garthwaite [22], who discussed the supply-side effect of public health insurance expansions in order to focus only on the program size and the size variation. Limwattananon et al. [23] also studied the supply-side reform of the medical system in Thailand.

## 3. Methodology

This study defined the carrying capacity of medical staff as the quantity of medical staff that a region can support through regional resources, such as population, income, healthcare expenditure, etc. The research condition herein is that each DMU necessarily faces the same measurement standard and that some input factors are not under control. Thus, we employed the output-oriented slack-based measure (SBM) model proposed by Tone [24,25] under the CRS framework.

Let the DMU set be *J* = {1, 2, ..., *n*}, and each DMU uses *m* kinds of input factor to produce *s* kinds of output in which the input vector and output vector for DMU *j* can be represented as **x***_j_* = (*x*_1*j*_, *x*_2*j*_, ..., *x_mj_*)^T^ and **y***_j_* = (*y*_1*j*_, *y*_2*j*_, ..., *y_sj_*)^T^. The input matrix and output matrix are then **X** = (**x**_1_, **x**_2_,..., **x***_n_*) ∈ *R^m^*^’*n*^ and **Y** = (**y**_1_, **y**_2_, ..., **y***_n_*) ∈ *R^s^*^’*n*^, where **X** > 0 and **Y** > 0. The production possibility set (PPS) for all DMUs is ***P*** = {(**x**, **y**) | **x** ≥ ∑*λ_j_***x***_j_*, **y** ≤ ∑*λ_j_***y***_j_*, ***λ*** ≥ 0}, where ***λ*** = (*λ*_1_, *λ*_2_, ..., *λ_n_*)^T^ represents the weight vector. In order to have powerful discrimination on the computation results and output-side research, we introduced the output-oriented SBM model with the CRS framework to assess the efficiency score of DMU *o* as follows:

(1) $$\begin{array}{c} \;\;\;\;\;\;\;\;\;{\rho_{o}}^{*}=\min\;\rho_{o}=1/[1+(1/s)\sum_{r=1}^{s}({s_{r}}^{+}/y_{ro})]\\ \mathrm{Subject}\mathrm{to}\\ x_{io}=\sum_{j=1}^{n}\lambda_{j}x_{ij}+{s_{io}}^{-},\\ y_{ro}=\sum_{j=1}^{n}\lambda_{j}y_{rj}-{s_{ro}}^{+}, \end{array}$$

where *λ_j_* ≥ 0, *s_io_*^-^ ≥ 0, *s_ro_^+^* ≥ 0, *i* = 1, 2,..., *m*, and *r* = 1, 2,..., *s*. The symbols *x_io_* and *y_ro_* respectively stand for the *i*^th^ input and *r*^th^ output for DMU *o*; the symbols *x_ij_* and *y_rj_* are the *i*^th^ input and *r*^th^ output for DMU *j*, respectively; the symbols *s_io_*^−^ and *s_ro_^+^* are slacks for the *i*^th^ input and *r*^th^ output for DMU *o*; the symbol *λ_j_* is the weight for DMU *j*. Here, ρ_o_^*^ = 1 means that DMU *o* has the best effective output and *s_ro_^+^* = 0 for “*r*; otherwise, ρ_o_^*^ < 1 means that DMU *o* has a more ineffective output and *s_ro_^+^* > 0 for “*r*, and ρ_o_^*^ ∈ [0, 1].

By using Model (1), we can find the target output on the *r*^th^ output for DMU *o* as y_r__o_^*^ (= *y_r__o_* + *s_r__o_^+^*), which is the summation of the actual output and the slack output. Based on the idea of the carrying capacity theory, the target output value y_r__o_^*^ is a measurement of the carrying capacity in which the current inputs can support the maximized output. In addition, we established the index of carrying efficiency (CE) to assess the achievement ratio of the carrying capacity. This study used DMU *o* on the *r*^th^ output as a case to present the index of carrying efficiency as follows:

CE*_ro_* = *y_r__o_* / y_r__o_^*^, (2)

where *y_r__o_* stands for the actual output for DMU *o* on the *r*^th^ output. Here, CE*_ro_* = 1 means that the *r*^th^ output of DMU *o* reaches the highest carrying capacity, because of *s_ro_^+^* = 0 and *y_r__o_* = y_r__o_^*^; CE*_ro_* < 1 means that the *r*^th^ output of DMU *o* still has potential carrying capacity, because of *s_ro_^+^* > 0 and *y_r__o_* < y_r__o_^*^, such that its carrying efficiency is lower than 1. In other words, higher carrying efficiency means that a DMU’s current outputs are close to its target values. Lower carrying efficiency implies that the DMU’s current outputs are lower than its target values. This index is available to compute by using the SBM model. Furthermore, *s_ro_^+^* and *y_ro_* can also be applied to compute a potential adjustment ratio (PAR) for the *r*^th^ output of DMU *o* by the index as follows:

PAR*_ro_* = (*s_ro_^+^* / *y_ro_*) × 100%, (3)

where PAR*_ro_* ∈ [0, 1]. The score of PAR*_ro_* being 0 means that the carrying efficiency is 1 and the DMU does not have any space to accommodate any other output, i.e., *s_ro_*^+^ = 0; on the contrary, PAR*_ro_* > 0 means that there is potential room for the DMU to increase output, i.e., *s_ro_*^+^ > 0.

The computations of carrying efficiency and potential adjustment ratio in Equations (2) and (3) come from the computation results from Model (1) in which we obtained the target output value (y_r__o_^*^) and the slack output (*s_r__o_^+^*). The applications of the target output value and the slack output allowed this study to obtain more additional information from a key issue.

## 4. Empirical Analysis

This section assesses and discusses the relative issues concerning the carrying capacity of medical staff in Taiwan under the output-oriented SBM model. The benefit of using the SBM model is that it can treat the case with multiple input and output factors in which two outputs, the numbers of physicians and nurses, were the focus in this study.

### 4.1. Data Sources

The data used in this study were collected from the National Statistics, R.O.C (Taiwan). The DMUs are 22 administrative regions in Taiwan, including New Taipei City, Taipei City, Taoyuan City, Taichung City, Tainan City, Kaohsiung City, Yilan County, Hsinchu County, Miaoli County, Changhua County, Nantou County, Yunlin County, Chiayi County, Pingtung County, Taitung County, Hualien County, Penghu County, Keelung City, Hsinchu City, Chiayi City, Kinmen County, and Lienchiang County. The data period was from 2013 to 2017. We referred to past literature, such as Wharrad and Robinson [19] and Sloan and Hsieh [21], to employ three kinds of inputs, including regional population (*x*_1_), regional income (*x*_2_), and local government expenditure on healthcare (*x*_3_), and used the research target to choose two kinds of outputs, including the number of physicians (*y*_1_), and the number of nurses (*y*_2_). The definition of the regional population is the number of registered permanent residents in an administrative region. Regional income is a multiplier of personal regular income and the number of registered permanent residents in an administrative region. The definition of local government expenditure on healthcare is the final amount for local government expenditures on environmental sanitation, public health and sanitation checks, epidemic prevention and control, and so on. The number of physicians is their quantity supplied, including Western medicine, Chinese medicine, and dentists in an administrative region, and the number of nurses is their quantity supplied, including registered nurses and registered professional nurses in an administrative region. In respect of the five variables, the local government can use inputs to create outputs. The first two inputs are indirect factors that need intermediate factors, such as the local government providing good housing and working environments to affect the regional population and income and then to absorb the medical staff and increase their carrying capacity, and the final input is a direct factor relative to the social welfare expenditure on healthcare to directly absorb medical staff and to directly increase their carrying capacity. This study applied the SBM model on the panel data of 22 DMUs that spanned the time path from 2013 to 2017. All monetary data were transformed into real data by using a GDP deflator, with 2011 as the base year.

### 4.2. Descriptive Statistics Analysis

Table 1 lists the results of the descriptive statistics in which the variable with the largest coefficient of variation (CV) is the number of physicians, followed by local government expenditure on healthcare. We can see that the top two variables with the largest CV are relative to Taiwan’s medical system. In the outputs, the CV for the number of physicians is larger than that for the number of nurses, implying that the problem of a physician maldistribution in Taiwan is more serious than that of nurses. In the inputs, the CV of the local government expenditure on healthcare is the largest of all the input factors, implying that Taiwan’s local governments should put forth a lot of effort to take care of the population in their administrative region, no matter whether the region has a high income or low income. Based on the CV measurement, the descriptive statistics analysis presents that the problem of a physician maldistribution in Taiwan is an important issue to research in order to find the target quantity of physicians in its administrative regions. The CV of the population does not indicate that Taiwan’s rural–urban disparity illustrates a harsh problem, but the problem of this rural–urban disparity is still valuable to investigate using the theory of carrying capacity.

Table 2 shows the correlation coefficient between the input and output variables. A positive relation present among the input and output variables means that increasing the input factors does not make the output factors decrease. Among all the input factors, regional incomes have the highest correlation coefficient with the numbers of physicians and nurses, denoting that the regional income strongly relates to the distribution of medical staff in Taiwan. Furthermore, the local government expenditure on healthcare exhibits a stronger association with the local distribution of physicians than that of nurses.

### 4.3. The Carrying Capacity of Medical Staff for Administrative Regions in Taiwan

**Figure 1:**
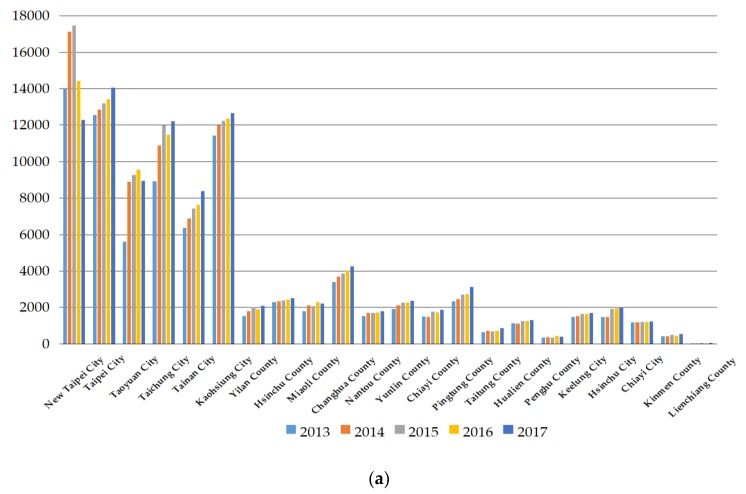

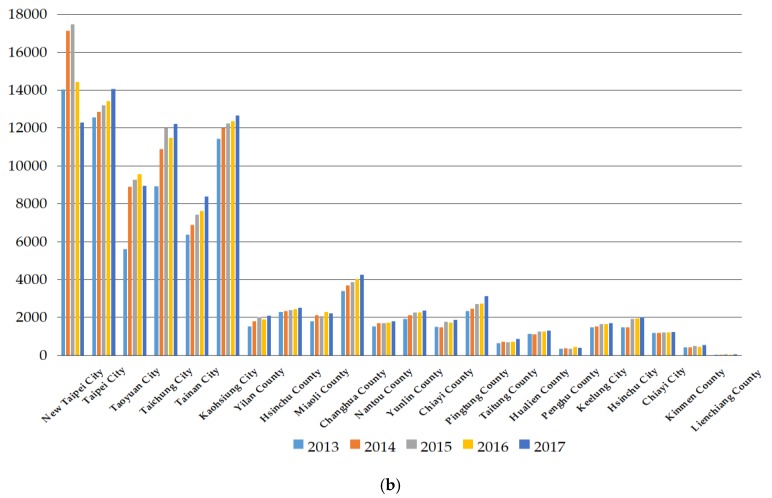
(**a**) Carrying capacity of physicians for administrative regions in Taiwan; (**b**) Carrying capacity of nurses for administrative regions in Taiwan.

We used the SBM model in Model (1), based on the inputs of each administrative region in Taiwan, to compute the target quantity of medical staff, including physicians and nurses, of each administrative region in Taiwan. The computation results are shown in Table A1 in the appendix Section. The information in Table A1 helped us to discuss the carrying capacity and to compute the carrying efficiency and the potential adjustment ratio of the carrying capacity. We translated the information in Table A1 into the following figures. Figure 1a presents the carrying capacity of physicians in 22 administrative regions of Taiwan from 2013 to 2017, showing that the six metropolises in Taiwan of New Taipei City, Taipei City, Taoyuan City, Taichung City, Tainan City, and Kaohsiung City own the top six carrying capacities of physicians. Among the six metropolises, Taipei City, Tainan City, and Kaohsiung City exhibit a continuous increase in the carrying capacity of physicians from 2013 to 2017. Changhua County is seventh in the carrying capacity of physicians in Taiwan but also increased from 2013 to 2017. Hsinchu County, Nantou County, Pingtung County, Keelung City, and Chiayi City also increased their carrying capacity of physicians from 2013 to 2017, and the percentage in the number of administrative regions with a continuous increase in the carrying capacity of physicians among the 22 administrative regions is 41%.

Figure 1b presents the administrative regions with a continuous increase in the carrying capacity of nurses from 2013 to 2017, including Taipei City, Tainan City, and Kaohsiung City, which are three of the top six metropolises in Taiwan. The trend of the carrying capacity of nurses in the other three metropolises is a movement up from 2013 to 2017, excluding New Taipei City, which is a neighbor to Taipei City; the latter is a higher urbanized city than the former. The likely reason that causes a continuously decreasing carrying capacity of medical staff in New Taipei City for 2016 and 2017 offers big potential to increase the number of medical staff and could thus become a strong magnet effect for Taipei City. Aside from them, Hsinchu County, Changhua County, Nantou County, Yunlin County, Pingtung County, Keelung City, and Chiayi City also exhibit a continuous increase in the carrying capacity of nurses from 2013 to 2017, and the percentage of administrative regions with a continuous increase in the carrying capacity of nurses among all 22 administrative regions in Taiwan is 45%.

Based on the results in Figure 1a,b, a continuous increase in the carrying capacities of physicians and nurses means that the administrative region has sufficient resources to support the greater numbers of physicians and nurses. Some past studies have concluded that the density of resources, such as population size, per capita incomes, per capita GDP, and so on, will cause the carrying capacity to vary [4,17,18,19,21]. We also found that the carrying capacity of nurses is always higher than that of physicians in Taiwan’s administrative regions. Toyabe [16] found that the shortage of physicians is also a key problem in Japan’s healthcare system. Penghu County, Kinmen County, and Lienchiang County are three outlying islands of Taiwan. Their carrying capacities of physicians and nurses always rank in the bottom three, implying that these regions are weak compared to other administrative regions in Taiwan and also the carrying capacities of physicians and nurses of these outlying islands are weaker than those on the main island of Taiwan. Hence, the Taiwan government can try to initiate greater resource input as a solution to spur the carrying capacity of medical personnel in the outlying islands. Kabene et al. [13] identified that high-quality healthcare depends on the development of new policies within human resources management for healthcare, and Tsai et al. [15] also concluded that a country with a large PD discrepancy needs to examine physicians’ workloads and their well-being. In general, the results in Figure 1a,b still show an increasing trend of the carrying capacity of medical staff in Taiwan’s medical system.

### 4.4. The Carrying Efficiency of Medical Staff for Administrative Regions in Taiwan

The carrying capacity is decided by the target value of medical staff, and an increase in the carrying capacity is not necessary for an increase in the carrying efficiency. We applied the idea of the achieve ratio proposed by Chang et al. [26] to assess the carrying efficiency of medical staff for administrative regions in Taiwan by using Equation (2). The computation results are shown in Table A2 in the appendix Section.

**Figure 2:**
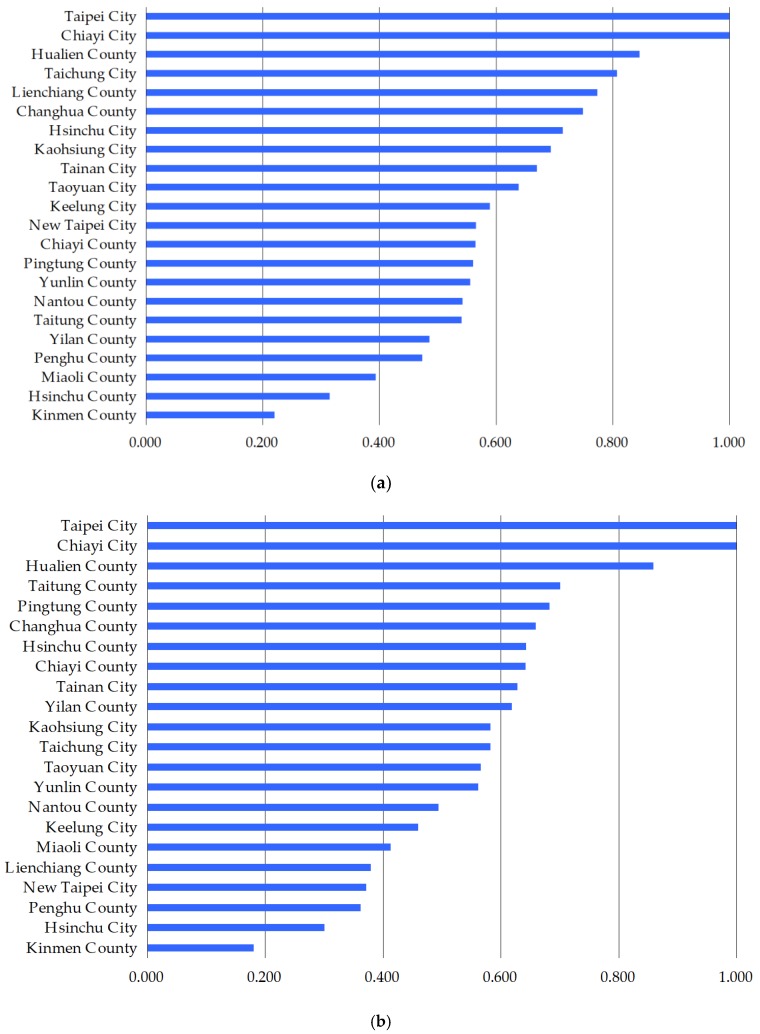
(**a**) Average carrying efficiency of physicians for administrative regions in Taiwan; (**b**) Average carrying efficiency of nurses for administrative regions in Taiwan.

This study used some information from Table A2 and rearranged it as Figure 2 in which if the carrying efficiency of physicians and nurses in Taipei City and Chiayi City are 1, then it means there is no more room for these two administrative regions in Taiwan to accommodate more physicians and nurses. Except for Taipei City and Chiayi City, the other administrative regions in Taiwan still have room to absorb more physicians and nurses since their carrying efficiencies are lower than the efficiency score of 1. The administrative regions with the lowest and the second lowest carrying efficiencies are Kinmen County and Hsinchu County, implying that these two administrative regions can accommodate a lot more physicians and nurses. In addition, the carrying efficiency of physicians in Miaoli County and the carrying efficiency of nurses in Penghu County are the third lowest, indicating that these two regions can accommodate more physicians and nurses, respectively. A low carrying efficiency, which denotes being able to accommodate more medical staff, is not a good phenomenon, because it indicates that the achievement ratio is low, and that the problem of maldistribution of medical staff has gotten worse. The problem for regions with a low carrying efficiency is that they fail to attract sufficient medical staff. Kinmen County, Hsinchu County, Miaoli County, and Penghu County have a low carrying efficiency of medical staff since they lack resources to attract more of them. Chang and Halfon [18] suggested the solution of a maldistribution of physicians is a proper manpower policy since they found that physicians in the United States always concentrate in states with a larger number of residency training positions and states with a high per capita income.

**Figure 3:**
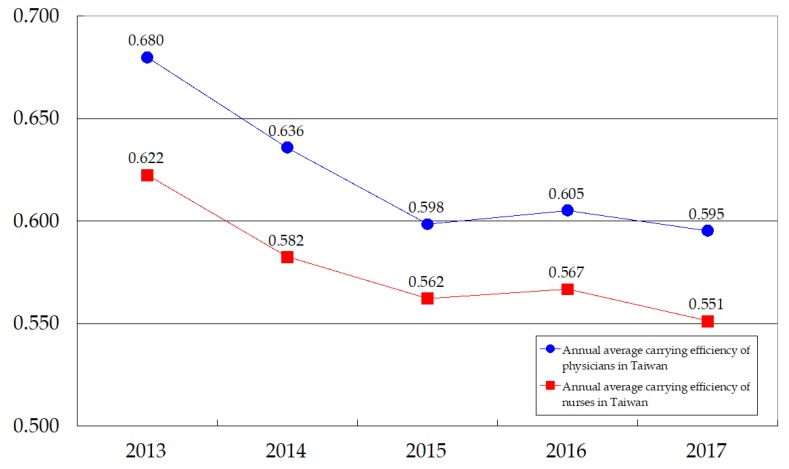
Annual average carrying efficiency of medical staff in Taiwan.

We next checked the carrying efficiency of medical staff for all of Taiwan by Figure 3 in which all administrative regions in Taiwan have the same weight since the medical system in Taiwan is indifferently emphasized in any administrative region. A surprise finding is that the trends of the carrying efficiency of physicians and nurses in Taiwan are decreasing, denoting that the central government should put more resources into improving the regional carrying efficiency of medical staff. Comparing between physicians and nurses, Taiwan’s carrying efficiency in the latter is lower than that in the former for each observation period. This result tells us that (i) Taiwan’s medical resource inputs are lacking and more resources should be put into the medical field; (ii) Taiwan placed more attention on physicians’ cultivation than on nurses’ cultivation in the past; and (iii) Taiwan should increase the supply of healthcare staff, especially nurses, as it becomes an aging society and eventually a super senior society. From this perspective, a lower carrying efficiency of nurses versus that of physicians is a warning for Taiwan’s current aged society and future super senior society. To cultivate more nurses, the Taiwan government should enhance the carrying efficiency of nurses by giving more resources to their cultivation. Wharrad and Robinson [19] and Sloan and Hsieh [21] explored the nurse issue and obtained a consistent conclusion that the GDP per capita significantly influences the global distribution of nurses. Based on their conclusion, increasing Taiwan’s GDP per capita may be another channel to absorb more foreign healthcare workers as a supplement for home-country nurses, because Taiwan is becoming an aged society.

### 4.5. An Analysis on the Potential Adjustment Ratio of Medical Staff

This section used Equation (3) to compute the potential adjustment ratio of administrative regions in Taiwan. The detailed computation result of this is shown in Table A3 in the appendix Section. Given the demand side, a 0% potential adjustment ratio means that the quantity of supplied medical staff has reached the target number; on the other hand, if the potential adjustment ratio is larger than 0%, then there is room for the region to increase the quantity of supplied medical staff, and it also means that the quantity of supplied medical staff has not reached the target quantity supplied and also that there is a shortage of medical staff.

**Figure 4:**
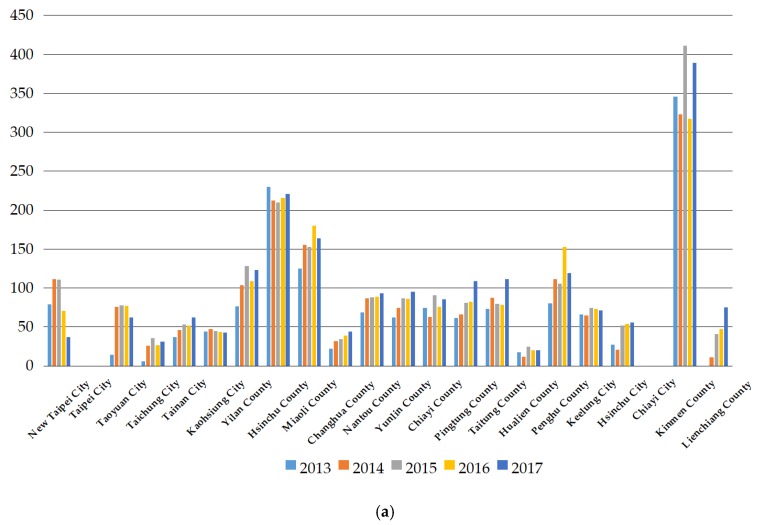

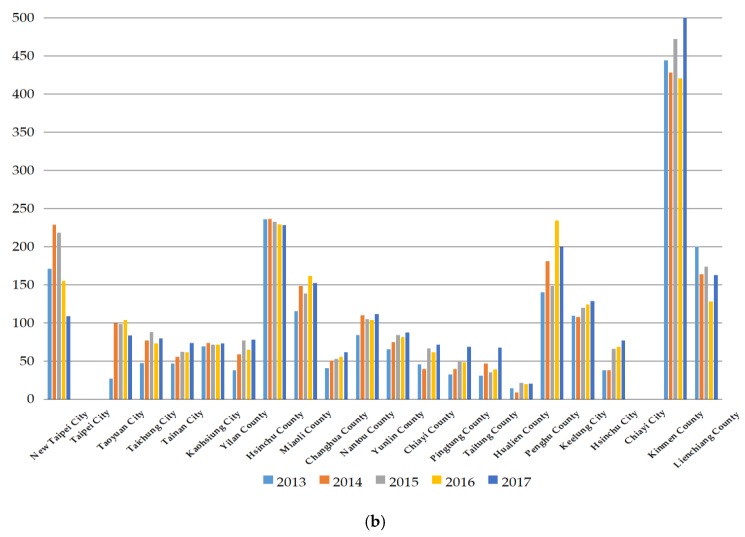
(**a**) The potential adjustment ratio of physicians for each administrative region in Taiwan; (**b**) The potential adjustment ratio of nurses for each administrative region in Taiwan.

Figure 4a shows the change in the potential adjustment ratio for each administrative region in Taiwan from 2013 to 2017. We found that the changes to the potential adjustment ratios in Changhua County, Nantou County, Pingtung County, and Lienchiang County are increasing year by year, meaning that a lack of resources in these regions makes them continue to experience an increased gap of physician slack. However, the regions with the top three values of physician slack from 2013 to 2017 are Kinmen County, Hsinchu County, and Miaoli County, implying that they lack resources the most in Taiwan to accommodate more physicians, and thus they have a great gap in their physician slack. The changes in potential adjustment ratios for Taipei City and Chiayi City are 0%, and their potential adjustment ratios remain at 0%, meaning that these two regions have sufficient resources to support the number of physicians without any physician slack. Hence, there is no potential adjustment on the number of physicians in Taipei City and Chiayi City.

Figure 4b shows that the region with the top potential adjustment ratio of nurses for all observation years is Kinmen County. This result implies that Kinmen County should inject more resources than other administrative regions in order to support the number of increasing nurses. It also implies that Kinmen County is the region that lacks nurses the most in Taiwan. On the other hand, Taipei City and Chiayi City have sufficient resources to support the current number of nurses. Hence, their potential adjustment ratios of nurses are 0%.

Based on the result in Figure 4b, the change of potential adjustment ratios in Changhua County is increasing year by year, which implies that the carrying capacities of nurses there is weakening year by year. Comparing Figure 4a with Figure 4b, we find that the changes in potential adjustment ratios of physicians and nurses in New Taipei City decreased after 2013, which means that New Taipei City has injected more resources than other administrative regions in Taiwan to cause the gaps of physicians and nurses to shrink after 2013. In addition, the gap of nurses supplied in Taiwan is always greater than that of the physicians supplied. Hence, the Taiwan government should place more attention on closing the gap between nurses and physicians supplied, such as injecting more resources into nurses’ cultivation over that of physicians’ cultivation. Given the demand side, this finding also shows that nurse leakage is more serious than physician leakage and that the nurse shortage is greater than the physician shortage. A serious lack in the number of nurses does not benefit the long-term development of Taiwan’s healthcare system when facing an aging society. Dussault and Franceschini [20] provided a suggestion to solve the maldistribution of medical staff and the shortage of healthcare workforce that includes equitable socioeconomic conditions between rural and urban areas, adequate investment in human resources, and stable and proper political institutions. Based on their suggestion, this study provides a similar recommendation, including balancing the regional population, increasing regional income, and subsidizing local governments’ expenditures on healthcare to solve the problems of maldistribution and shortage in the healthcare workforce.

## 5. Conclusions

This study used an analysis of the carrying capacity and carrying efficiency to discuss the target quantities of physicians and nurses within the administrative regions of Taiwan by the SBM model in order to find a solution for the maldistribution of medical staff and a way to adjust their workforce. Although the quantity supplied of medical staff in Taiwan is increasing year by year, this increased quantity is very concentrated in a few administrative regions of Taiwan instead of being dispersed on average to all administrative regions, thus causing the phenomenon of a maldistribution of medical staff around the country. Hence, the carrying capacity and the carrying efficiency of medical staff is rather distinguishable. The idea of carrying capacity relates to a target value, the idea of carrying efficiency relates to an achievement ratio, and a high (low) carrying capacity is not necessary for a high (low) carrying efficiency. Generally speaking, a region with sufficient resources will exhibit a high carrying capacity, high carrying efficiency, and a low potential adjustment ratio. Conversely, a region with insufficient resources has a low carrying capacity, low carrying efficiency, and a high potential adjustment ratio.

This study used 22 administrative regions in Taiwan as a research sample and concluded that Taipei City and Chiayi City present sufficient resources to support their high carrying efficiency of medical staff and low potential adjustment ratio on the number of medical staff. The carrying efficiency of medical staff and the potential adjustment ratio on the number of medical staff in Kinmen County and Hsinchu County are the bottom two, respectively, meaning the central government does not inject enough resources to support these two counties. Kinmen County is one of the few outlying islands of Taiwan, with the other two being Penghu County and Lienchiang County. The carrying efficiencies and the potential adjustment ratios in these three outlying islands of Taiwan are relatively poorer than other administrative regions on the main island of Taiwan. This denotes that the Taiwan government’s resource support between the main island and the outlying islands has a gap. Except for Taipei City and Chiayi City, the other administrative regions in Taiwan have room to upgrade their carrying efficiencies of medical staff and increase the number of medical staff. First, the room for improvement in nurses is larger than that in physicians. Shrinking the gap between nurses and physicians, upgrading the carrying capacity and carrying efficiency of medical staff, increasing the number of medical staff, and balancing the distribution of medical staff all needs governmental resource inputs for support.

Based on a given demand side, we offer some policy suggestions to the Taiwan government as follows: (i) Taiwan has the phenomenon in the medical field that physicians generally concentrate in a wealthy region, and that places with a greater population always present a higher supply of nurses. Hence, the Taiwan government should initiate different policies to spur the carrying capacities and carrying efficiencies of physicians and nurses. (ii) The government should pay more attention to cultivating more nurses since the problem of their shortage is more serious than the same problem for physicians. This is very important, as a shortage of nurses does not benefit the long-term development of Taiwan’s healthcare system when facing a looming aging society. (iii) Taiwan’s medical resource inputs are lacking, and more resources should be put into the medical field in order to upgrade the carrying capacity and carrying efficiency of medical staff, to increase the number of medical staff, to shrink the gap between nurses and physicians, and to balance the distribution of medical staff. (iv) The maldistribution of medical staff also creates rural–urban disparity, and there is a maldistribution of medical staff between Taiwan’s main island and its outlying islands. Based on the medical problems above, the Taiwan government should place more effort on medical resource inputs and distribution. For strong discrimination on the carrying capacity score in an empirical analysis, this study considered three input variables and two output variables. Future research can consider the addition of more inputs and choose another DEA model that maintains strong discrimination on the empirical result in order to obtain more information and implications.

## Figures and Tables

**Table 1:** Descriptive statistics of input and output variables.

Variable		Unit	Standard Deviation	Mean	Coefficient of Variation
Inputs	Population (*x*_1_)	Persons	1,087,526	1,067,367	1.019
	Regional income (*x*_2_)	NT$10 million	52,798	45,092	1.171
	Local government expenditure on healthcare (*x*_3_)	NT$10 million	105	86	1.221
Outputs	No. of physicians (*y*_1_)	Persons	3568	2904	1.229
	No. of nurses (*y*_2_)	Persons	7222	6773	1.066

Note: All currency variables are deflated into real ones at the 2011 price level by Taiwan’s GDP deflators.

**Table 2:** The correlation coefficient between input and output variables.

Outputs/Inputs	Population (*x*_1_)	Regional Income (*x*_2_)	Local Government Expenditure on Healthcare (*x*_3_)
No. of physicians (*y*_1_)	0.916	0.957	0.917
No. of nurses (*y*_2_)	0.919	0.941	0.896

## Appendix A

**Table A1:** Target quantity of medical staff for each administrative region in Taiwan.

DMUs	2013		2014		2015		2016		2017	
	Physician	Nurse	Physician	Nurse	Physician	Nurse	Physician	Nurse	Physician	Nurse
New Taipei City	14,032	39,747	17,119	48,428	17,462	49,507	14,429	41,613	12,284	36,001
Taipei City	12,555	24,136	12,841	24,409	13,188	25,467	13,422	26,270	14,056	27,381
Taoyuan City	5591	15,838	8883	25,129	9261	26,255	9542	27,520	8936	26,188
Taichung City	8919	25,264	10,876	30,766	11,984	33,978	11,476	33,098	12,199	35,752
Tainan City	6355	18,001	6880	19,464	7423	21,046	7608	21,943	8377	24,550
Kaohsiung City	11,421	32,351	11,993	33,927	12,221	34,648	12,348	35,613	12,648	37,067
Yilan County	1509	4274	1799	5090	2015	5712	1897	5470	2080	6095
Hsinchu County	2282	6463	2320	6564	2384	6758	2432	7015	2515	7371
Miaoli County	1797	5091	2098	5936	2060	5841	2289	6602	2210	6478
Changhua County	3385	9589	3677	10,403	3859	10,942	4023	11,603	4254	12,466
Nantou County	1527	4325	1689	4778	1696	4809	1708	4926	1800	5276
Yunlin County	1906	5400	2111	5973	2266	6424	2254	6500	2352	6893
Chiayi County	1496	4239	1482	4191	1765	5004	1715	4946	1856	5440
Pingtung County	2337	6621	2446	6920	2690	7625	2724	7855	3108	9110
Taitung County	630	1784	705	1993	682	1933	704	2030	851	2493
Hualien County	1138	3225	1099	3110	1253	3551	1244	3588	1295	3797
Penghu County	329	933	365	1032	336	953	440	1268	387	1135
Keelung City	1470	4163	1515	4285	1636	4640	1653	4768	1692	4958
Hsinchu City	1482	4197	1482	4192	1909	5412	1943	5604	2009	5888
Chiayi City	1165	3300	1169	3307	1189	3371	1199	3458	1227	3596
Kinmen County	419	1186	418	1183	480	1362	442	1275	533	1561
Lienchiang County	31	84	30	84	34	96	32	93	37	108
Total	81,777	220,211	92,998	251,166	97,793	265,334	95,525	263,060	96,705	269,603

**Table A2:** The carrying efficiency of medical staff for each administrative region in Taiwan.

DMUs	2013		2014		2015		2016		2017	
	Physician	Nurse	Physician	Nurse	Physician	Nurse	Physician	Nurse	Physician	Nurse
New Taipei City	0.560	0.369	0.474	0.305	0.475	0.314	0.588	0.393	0.732	0.480
Taipei City	1.000	1.000	1.000	1.000	1.000	1.000	1.000	1.000	1.000	1.000
Taoyuan City	0.876	0.790	0.570	0.502	0.563	0.505	0.565	0.490	0.619	0.545
Taichung City	0.949	0.680	0.795	0.566	0.738	0.532	0.790	0.578	0.766	0.557
Tainan City	0.731	0.684	0.688	0.643	0.654	0.618	0.661	0.620	0.619	0.576
Kaohsiung City	0.695	0.592	0.681	0.577	0.692	0.584	0.700	0.584	0.701	0.578
Yilan County	0.567	0.727	0.492	0.630	0.439	0.566	0.480	0.608	0.449	0.563
Hsinchu County	0.303	0.298	0.320	0.297	0.323	0.301	0.317	0.304	0.312	0.305
Miaoli County	0.446	0.465	0.392	0.403	0.396	0.419	0.358	0.383	0.379	0.397
Changhua County	0.823	0.713	0.760	0.665	0.745	0.655	0.721	0.643	0.697	0.620
Nantou County	0.594	0.543	0.536	0.477	0.532	0.488	0.532	0.491	0.518	0.474
Yunlin County	0.619	0.606	0.574	0.573	0.536	0.544	0.539	0.552	0.512	0.535
Chiayi County	0.574	0.688	0.615	0.718	0.525	0.602	0.571	0.621	0.540	0.584
Pingtung County	0.620	0.757	0.603	0.716	0.553	0.672	0.550	0.675	0.479	0.594
Taitung County	0.578	0.765	0.535	0.683	0.557	0.740	0.561	0.720	0.474	0.598
Hualien County	0.855	0.879	0.899	0.923	0.805	0.825	0.834	0.835	0.835	0.832
Penghu County	0.556	0.417	0.474	0.356	0.488	0.403	0.396	0.300	0.457	0.334
Keelung City	0.603	0.478	0.607	0.482	0.574	0.456	0.579	0.446	0.585	0.438
Hsinchu City	0.786	0.725	0.831	0.726	0.659	0.603	0.652	0.593	0.644	0.566
Chiayi City	1.000	1.000	1.000	1.000	1.000	1.000	1.000	1.000	1.000	1.000
Kinmen County	0.224	0.184	0.237	0.189	0.196	0.175	0.240	0.192	0.205	0.167
Lienchiang County	1.000	0.333	0.905	0.379	0.710	0.365	0.679	0.439	0.572	0.381
Total	0.680	0.622	0.636	0.582	0.598	0.562	0.605	0.567	0.595	0.551

**Table A3:** The potential adjustment ratio (%) of medical staff for each administrative region in Taiwan.

DMUs	2013		2014		2015		2016		2017	
	Physician	Nurse	Physician	Nurse	Physician	Nurse	Physician	Nurse	Physician	Nurse
New Taipei City	78.73	170.91	111.03	228.39	110.41	218.09	70.09	154.72	36.55	108.40
Taipei City	0.00	0.00	0.00	0.00	0.00	0.00	0.00	0.00	0.00	0.00
Taoyuan City	14.15	26.62	75.44	99.04	77.58	98.15	76.90	103.88	61.53	83.33
Taichung City	5.41	47.07	25.83	76.56	35.48	87.85	26.56	72.94	30.53	79.42
Tainan City	36.81	46.30	45.43	55.46	52.84	61.86	51.32	61.21	61.65	73.52
Kaohsiung City	43.95	68.83	46.79	73.34	44.41	71.33	42.82	71.29	42.67	72.98
Yilan County	76.26	37.51	103.09	58.67	127.65	76.68	108.21	64.47	122.66	77.64
Hsinchu County	229.71	235.38	212.27	236.42	209.18	232.27	215.07	228.88	220.37	228.16
Miaoli County	124.39	115.19	154.98	148.28	152.47	138.50	179.51	161.37	163.77	151.87
Changhua County	21.51	40.21	31.57	50.37	34.20	52.70	38.63	55.53	43.56	61.32
Nantou County	68.34	84.12	86.44	109.77	87.85	104.90	88.10	103.64	93.17	111.14
Yunlin County	61.56	65.04	74.36	74.55	86.64	83.75	85.49	81.06	95.17	87.05
Chiayi County	74.20	45.36	62.63	39.24	90.38	66.12	75.16	61.10	85.23	71.33
Pingtung County	61.19	32.18	65.85	39.58	80.75	48.82	81.94	48.05	108.76	68.33
Taitung County	73.01	30.79	86.87	46.33	79.38	35.14	78.20	38.95	111.06	67.30
Hualien County	17.01	13.79	11.28	8.29	24.15	21.25	19.87	19.77	19.73	20.23
Penghu County	80.02	139.88	110.95	180.55	105.04	148.28	152.72	233.74	118.71	199.34
Keelung City	65.87	109.08	64.65	107.52	74.09	119.47	72.75	124.05	70.89	128.39
Hsinchu City	27.18	37.88	20.28	37.76	51.74	65.71	53.36	68.64	55.27	76.62
Chiayi City	0.00	0.00	0.00	0.00	0.00	0.00	0.00	0.00	0.00	0.00
Kinmen County	345.44	444.06	322.56	428.32	411.02	472.22	317.11	420.47	388.73	500.48
Lienchiang County	0.00	200.06	10.45	163.63	40.79	173.70	47.20	127.79	74.85	162.47
Total	68.40	90.47	78.31	102.82	89.82	108.03	85.50	104.62	91.13	110.42
